# Supplementary material for: Increasing proportion of mildly aged population in rural mitigates farmland abandonment in the farming-pastoral ecotone of northern China
Source: PLoS One. 2025 Jul 31;20(7):e0328483. doi: 10.1371/journal.pone.0328483 (PMC12312902; doi:10.1371/journal.pone.0328483)
Supplement: S1 Text — (DOCX) [file pone.0328483.s001.docx]

**S1 Text. Identification process of farmland abandonment**

The first step involves extracting annual farmland pixels from the CLCD and CACD datasets, and obtaining their intersection as the final farmland data in land use, thereby reducing mapping errors. Since the primary land use type in the region is grassland, the non-intersecting was revised to grassland. Meanwhile, we also extracted annual grassland and bare land pixels from the CLCD dataset, and ultimately obtaining a new land use type dataset from 1999 to 2022. In addition, due to insufficient monitoring data for three consecutive years in 2020, we simulated the land use type data for 2023 by duplicating the land cover data from 2022.

The second step involves using the 1999 farmland base map as a reference and applying a five-year sliding time window with identification rules to determine abandoned farmland after 2000. For example, if a pixel is classified as farmland in 1999, we define it as “abandoned farmland in 2000” if it becomes grassland or bare land in 2000 and remains grassland or bare land for the subsequent three years. To avoid misclassification of pixels corresponding to farmland returned to forests, topographic data were also used to remove pixels with slopes greater than 25°, resulting in the final abandoned farmland map.
