# Supplementary figures and images for: Increasing proportion of mildly aged population in rural mitigates farmland abandonment in the farming-pastoral ecotone of northern China
Source: PLoS One. 2025 Jul 31;20(7):e0328483. doi: 10.1371/journal.pone.0328483 (PMC12312902; doi:10.1371/journal.pone.0328483)

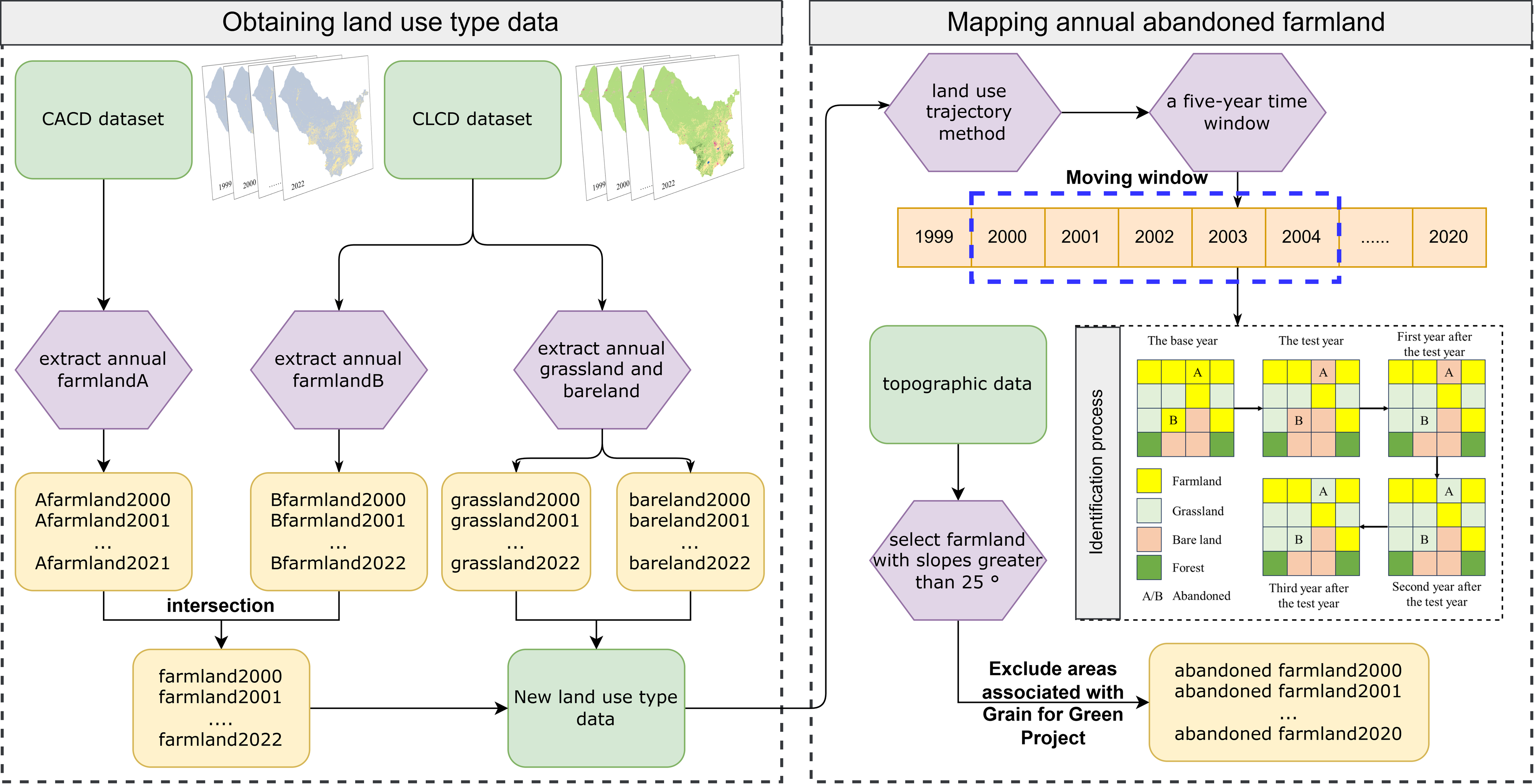

Supplement: S1 Fig — All map boundary data in this figure are consistent and publicly available from the National Platform for Common Geospatial Information Services (www.tianditu.gov.cn). The review map number is GS (2024) 0650. (TIF) [file pone.0328483.s002.tif]
