# Supplementary material for: Increasing proportion of mildly aged population in rural mitigates farmland abandonment in the farming-pastoral ecotone of northern China
Source: PLoS One. 2025 Jul 31;20(7):e0328483. doi: 10.1371/journal.pone.0328483 (PMC12312902; doi:10.1371/journal.pone.0328483)
Supplement: S2 Text — (DOCX) [file pone.0328483.s003.docx]

**S2 Text. Accuracy verification method of random forest**

Accuracy, recall, and precision are used as evaluation metrics to evaluate the accuracy of model predictions. The basis for accuracy evaluation involves dividing the sample set into training and test subsets using the stratified sampling method, and comparing the predicted values of the test subset with their observed values [1, 2]. In this study, 80% of the samples were used for modeling, and 20% for validation.

Accuracy refers to the proportion of samples correctly predicted by the model, describing the proportion of correctly classified samples in the total sample set. Recall (TPR) is the ratio of samples correctly identified as positive by the model to the total number of actual positive samples, which measuring the model’s ability to correctly identify positive categories. Precision is the proportion of actual positive samples among all samples predicted as positive by the model, measuring the accuracy of the model in predicting positive categories. The calculation formulas are as follows:

$Accuracy= \frac{TP+TN}{TP+TN+FP+FN}$ $( AUTONUM \backslash* Arabic )$

$Recall= \frac{TP}{TP+FN}$ $( AUTONUM \backslash* Arabic )$

$Precision= \frac{TP}{TP+FP}$ $( AUTONUM \backslash* Arabic )$

where TP refers to true positives, TN to true negatives, FP to false positives, and FN to false negatives.

**References**

1. Foody GM. Status of land cover classification accuracy assessment. Remote sensing of environment. 2002;80(1):185-201. doi: 10.1016/S0034-4257(01)00295-4.

2. Maxwell AE, Warner TA, Guillén LA. Accuracy assessment in convolutional neural network-based deep learning remote sensing studies—Part 1: Literature review. Remote Sensing. 2021;13(13):2450. doi: 10.3390/rs13132450.
