# Supplementary material for: Increasing proportion of mildly aged population in rural mitigates farmland abandonment in the farming-pastoral ecotone of northern China
Source: PLoS One. 2025 Jul 31;20(7):e0328483. doi: 10.1371/journal.pone.0328483 (PMC12312902; doi:10.1371/journal.pone.0328483)
Supplement: Attachment — Submitted filename: 1_1Response to Reviewers.docx [file pone.0328483.s005.docx]

**Subject: Revision Notes (Manuscript No: PONE-D-25-04045)**

Dear Editor

PLOS One

Thank you for the opportunity to revise our Manuscript **No. PONE-D-25-04045[Title: Increasing proportion of mildly aged population in rural mitigates farmland abandonment in the farming-pastoral ecotone of northern China]**. We are grateful for the detailed comments and suggestions provided by each of the reviewers, and we have thoughtfully taken into account these comments.

We sincerely apologize for the inconvenience caused by the previous version of the manuscript. Due to our oversight, the uncorrected original manuscript was inadvertently submitted, resulting in numerous errors in the figures. We have now uploaded the corrected version and kindly ask you to review it at your convenience. We apologize for any confusion or disruption this may have caused. Additionally, we have added more contact information in the “Data Availability” file to support the long-term accessibility and oversight of the data.

Below, we provide a detailed response to each of the editorial comments and outline the revisions or explanations accordingly.

**1. Format modification**

We have modified the file format and prepared the following revised materials according to PLOS ONE requirements:

1)Response to Reviewers (this document)

2)Revised Manuscript with Track Changes

3)Clean Manuscript (without track changes)

4)Ethics Statement Document and Data availability (uploaded under “Other” files)

5)Cover letter (adding the funding statement)

**2.** **Funding Statement**

As requested, we have added the following standard disclaimer to the cover letter: “The funders had no role in study design, data collection and analysis, decision to publish, or preparation of the manuscript.”

**3. Data Availability**

We regret that we are unable to share the full dataset used in this study due to confidentiality agreements and the need to protect the geospatial privacy of local residents. These restrictions are in compliance with local data governance regulations.

However, all openly available data sources used in the study have been clearly listed in the manuscript. Further explanation regarding the data sharing constraints and the contact information for data requests has been added to the Data Availability statement.

**4. Ethics Approval**

We confirm that our study does not require ethics approval, as it does not involve human or animal subjects, and no personal or sensitive information was collected. To comply with the editorial request, we have obtained a formal explanation letter from our affiliated institution—College of Land Science and Technology, China Agricultural University.

The statement was reviewed and approved by:

1) Dr. Haishuang Jia, Director of Scientific Research and Social Services,
2) Ms. Zhaohong Cao, Deputy Party Secretary and Vice Dean,
3) Ms. Xiaohe Yu, Chief Officer of General Affairs.

We have uploaded the scanned letter, including the official stamp in English version, under “Other” files for your reference. Additionally, the original manuscript included the ethical statement within the questionnaire data section. To comply with the requirements, we have relocated the ethics statement to the “Methods” section of the manuscript, where it is now clearly and separately stated (lines 307–315).

**5. Map and Satellite Image Copyright**

We have addressed the copyright concerns in the following ways:

1)Figure 3: The original basemap has been removed.

2)Figures 3, 4, and 5: All geographic boundary data (including China’s administrative boundaries) were obtained from National Geomatics Center of China, a publicly accessible and free source. The satellite images in Figure 5b also have been removed.

We have updated the explanatory section following the image title to clearly indicate the data source and have included the map approval number GS (2024) 0650 in the manuscript.

**6. Supporting information**

We have revised and improved the Supporting Information section as requested. Titles have been added to all Supporting Information files and listed at the end of the manuscript.

**7. Reference list**

We have revised and improved the reference format in the main text and appendices as requested, and added or removed certain references based on the reviewer's suggestions and their relevance to the main content of the manuscript. The newly added references have been highlighted in red.

Due to revisions in the Introduction and Discussion sections, we have added three new references to enhance the theoretical foundation and contextual relevance of the manuscript. The details of the added references are as follows:

[2.	Zheng QM, Ha T, Prishchepov AV, Zeng YW, Yin H, Koh LP. The neglected role of abandoned cropland in supporting both food security and climate change mitigation. Nature Communications. 2023;14(1):6083. doi: 10.1038/s41467-023-41837-y. (Line 561-563)

8.	Li S, Li X. The mechanism of farmland marginalization in Chinese mountainous areas: Evidence from cost and return changes. Journal of Geographical Sciences. 2019; 29:531-48. (Line 577-578)

71.	Ye J, Hu Y, Feng Z, Zhen L, Shi Y, Tian Q, et al. Monitoring of Cropland Abandonment and Land Reclamation in the Farming–Pastoral Zone of Northern China. Remote Sensing. 2024;16(6):1089. (Line 743-744)]

Considering the comments of reviewers, we have deleted certain sections of the manuscript and removed the corresponding references to maintain coherence and relevance.

[68.	Figueiredo J, Pereira HM. Regime shifts in a socio-ecological model of farmland abandonment. Landscape Ecology. 2011;26(5):737-49. doi: 10.1007/s10980-011-9605-3. PubMed PMID: WOS:000291485100011. (in the original manuscript)

71.	Han X, Wei C, Cao GY. Aging, generational shifts, and energy consumption in urban China. Proceedings of the National Academy of Sciences of the United States of America. 2022;119(37):e2210853119. Epub 2022/09/07. doi: 10.1073/pnas.2210853119. PubMed PMID: 36067298; PubMed Central PMCID: PMCPMC9478673. (in the original manuscript)

72.	Deng X, Zeng M, Xu DD, Qi YB. Why do landslides impact farmland abandonment? Evidence from hilly and mountainous areas of rural China. Natural Hazards. 2022;113(1):699-718. doi: 10.1007/s11069-022-05320-z. PubMed PMID: WOS:000776420900003. (in the original manuscript)]

We also deleted a duplicate reference: [38.	Wei Y, An P, Jin Y, Chen X, Zhang G, Pan Z. Population aging and its farmland effect on abandonment in the northern farming-pastoral ecotone: A case study of Ulanqab. Journal of Arid Land Resources and Environment. 2021;35(07):64-70. (in the original manuscript)]

**8. Supplementary explanation**

In response to the suggestions of editors and reviewers, we have made revisions in multiple sections of the manuscript. Additionally, during the revision process, we identified and corrected some grammatical and expression-related issues present in the original version. All modifications have been highlighted in red within the manuscript for ease of review.

Hopefully our responses and revisions are clear and meet the requirement of editor and reviewers. Moreover, please feel free to contact us if additional information is needed. We hope that all these changes fulfil the requirements to make the manuscript acceptable for publication in “PLOS One”.

Sincerely yours,

Dr. Pingli An

College of Land Science and Technology

China Agricultural University, China

List of Responses to Reviewers’ comment

**Reviewer #1**

**Comment 1**：When presenting “Spatio-temporal changes of abandoned farmland”, please tell use changes in the ratio of land abandonment, in addition to changes in the area of land abandonment.

***[Answer: Thanks for your constructive comment and this is indeed something worth adding. we have revised and improved the manuscript by further supplemented the changes in the abandonment ratio within the section on spatio-temporal changes of abandoned farmland (Line 319-333).*** ***Additionally, due to copyright considerations, we have redrawn the original Fig. 5. The detailed revisions are as follows:***

***“Meanwhile, the region exhibited a significantly increasing trend of grassland from 436,12 km^2^ to 453,83 km^2^, with a net gain of 177.1 km^2^ (Fig 5a). Notably, although the absolute area of abandoned farmland declined slightly from 295 km^2^ in 2000 to 273 km^2^ in 2020, the abandonment rate increased slightly from 3.03% to 3.66%.***

***Specifically, the temporal evolution of farmland abandonment can be broadly divided into three phases. The first phase (2000-2006) was the peak period of abandonment. The area of farmland abandonment reached a historical high of approximately 562 km^2^ in 2002, with a corresponding abandonment rate of 6.12%. In 2005, the abandonment rate reached its peak of 6.47%. The second phase (2007-2012) showed an overall downward trend. Both the abandonment area and rate declined and remained at relatively low levels during this period. The lowest values were observed in 2011, with 194 km^2^ of abandoned farmland and an abandonment rate of 2.42%. The third phase (2013-2020) remained at a moderate level but a slight decline. Although the abandoned area and abandonment rate rebounded in 2015, with 472 km^2^ of abandoned farmland and an abandonment rate of 5.47%, the subsequent continuous downward trend was significant, and by 2019, the abandoned area and abandonment rate had dropped to 239 km^2^ and 2.75%.” (Line 319-333)***

**Fig 5. Spatiotemporal pattern of abandonment from 2000 to 2020 *(Line 342)]***

**Comment 2：**I think there is no need to present the different behavioral patterns across different age groups in Lines 318-339, as it is not particularly relevant to the core content (land abandonment) of the manuscript.

***[Answer: Thank you very much for highlighting this issue. After careful consideration, we agree that the different behavioral patterns across different age groups was somewhat redundant, and we have therefore removed it from the manuscript. However, the section on basic household information remains relevant and important, as it reflects key characteristics of the surveyed households and*** ***also to some extent displays the relevant information of the questionnaire. To improve clarity and coherence, we have integrated this content into*** ***factors influencing farmers’ willingness to abandon farmland in Ulanqab Specifically, we have reorganized the section into two parts: (1) basic information of households of different age groups (Line 351), and (2)*** ***Comparative analysis of factors affecting farmland abandonment among households of different age groups (Line 362-363).]***

Comment 3：“Mechanism of rural population aging on farmland abandonment” should be the content in Results.

***[Answer: Thank you very much for pointing out it. We fully agree with your suggestion. Accordingly, and in consideration of the overall structure of the manuscript, we have made the following adjustments:*** ***we have relocated part of the original content titled “Mechanism of rural population aging on farmland abandonment” to the Results section, specifically in the second paragraph, to better emphasize the core theme of our research.*** ***The remaining content has been retained in the Discussion section but has been supplemented and reorganized to focus on other relevant factors influencing farmland abandonment in the context of population aging.*** ***Specifically, the revised content has been divided into two sections:***

***(1) Mechanism of rural population aging on farmland abandonment (Results, Line 435-452). The detailed revisions are as follows:***

***“At the regional level, a random forest model was further employed to simulate scenarios where the proportion of the mildly aged population (60-69) remained unchanged, while the proportion of the severely elderly population increased by 5% and 10%, respectively. The results revealed that the abandonment rate of farmers increased to 24.4% and 34.0%, respectively. These findings suggested that as the severely aged population increases, the agricultural labor supply becomes increasingly constrained, thereby intensifying the risk of land abandonment.” (Results, Line 437-442)***

***“At the household level, population aging is reflected in the increase in the average age of family members and the decrease in the number of agricultural laborers. Empirical evidence confirms that the average age of households and the number of agricultural laborers were key determinants of farmland abandonment highlighting the complex impact mechanism of rural population aging on farmland abandonment.” (Results, Line 448-452)***

***(2) Driving factors of farmland abandonment in Ulanqab (Discussion, Line 454-472). The detailed revisions are as follows:***

***“Identifying the driving factors influencing farmland abandonment is a complex process. Based on the regional characteristics of deep rural population aging, this study focuses on analyzing the impact of rural population aging on farmland abandonment from both regional and household perspectives, thereby effectively supplementing the limited attention currently given to farmland abandonment in relation to changes in population age structure [4, 68]. Moreover, Previous studies have identified various determinants of abandonment, including land type, land quality, number of farm laborers, the average age of farm laborers and farmland area per capita [4, 17, 18, 36, 38], which is consistent with our research findings.” (Discussion, Line 455-462)***

***“Therefore, in the future, more thoroughly natural and socio-economic factors should be integrated to explore the compound driving mechanisms of farmland abandonment under the context of population aging and then provide a scientific foundation for more targeted policy interventions and the optimization of land use strategies.” (Discussion, Line 469-472)]***

**Comment 4：**I did not see the necessity of Table 6 in the manuscript.

***[Answer: Thank you very much for your suggestion.*** ***After careful reconsideration, we also agree that Table 6 is somewhat redundant and does not substantially enhance the overall findings. Therefore, we have removed Table 6 from the manuscript to improve clarity and conciseness.]***

**Comment 5：**All images have a lower resolution, which makes reading difficult. Figure 1 is too complicated to understand. It is recommended to retain only the core elements. Figure 7 is also not necessary.

***[Answer:*** ***Many thanks for your suggestion. We sincerely apologize for the low resolution of the images and some of the images being too complex in our manuscript.*** ***In response, we have enhanced the resolution of all figures to ensure a clearer visual presentation. Meanwhile, we have simplified Figure 1 to improve its readability and supplemented missing elements in Figure 2. Additionally, after careful consideration, we concluded that Figure 7 does not contribute substantially to the manuscript and have therefore removed it. The detailed descriptions are as follows:***

***Fig. 1.*** ***Theoretical analysis framework for household farmland-use behavior (The theoretical framework and research hypotheses, Line 123)***

***Fig. 2. Household type and farmland abandonment mechanism. (The theoretical framework and research hypotheses, Line 161)]***

**Reviewer #2**

**Comment 1：**Your topic is “The Increase in the proportion of lightly elderly people in Rural areas alleviates the abandonment of farmland in the Agro-pastoral Ecotone of Northern China”. It should focus on this theme. Other aspects such as the prediction of abandonment are your auxiliary and supplementary analysis. Please grasp this key theme throughout the text.

***[Answer: Thank you very much for your suggestion. We deeply recognize that our initial description of the central theme was insufficient. Therefore, we further strengthen our grasp of the key theme of this manuscript.*** ***Specifically, for example, in “Changes in Population and Abandoned Farmland from 2020 to 2100” section, our original intention was to use future projections of farmland abandonment to reveal the distinct impacts of changes in the mildly and severely aged population structure. However, we acknowledge that our previous expression lacked precision and did not effectively highlight this objective.*** ***To address this, we have revised the section by removing irrelevant content and enhancing the emphasis on how variations in the proportions of mildly and severely aged populations affect farmland abandonment. These revisions aim to more clearly convey the central theme of the study—understanding the relationship between population aging and farmland abandonment. (see Changes in population and farmland abandonment from 2020 to 2100, Line 414-419, 426-430). The detailed descriptions are as follows:***

***“From 2010 to 2100, differences in the age structure of population were observed under different scenarios. In the SSP1 and SSP5 scenarios, the age structure of the population in 2100 is nearly identical, with slight differences in the proportion of population over 70 years old, at 38.91% and 38.89%, respectively. In the SSP2 scenario, the proportion of population aged 60-69 is 11.35%, and the proportion of those aged 70 and above is 27.29%, both of which are lower than in the other two scenarios (Fig 6a). Moreover, the population of Inner Mongolia presented a trend of first rising and then declining under different scenarios (Fig 6b).” (Line 414-419)***

***“These scenarios suggested that a decreasing of the mildly aged population and an increasing of the severely aged population is likely to exacerbate farmland abandonment. In contrast, in the SSP2 scenario, the increase in the proportion of the population aged 70 and above was lower than in the other two scenarios, with the abandonment proportion at 20.1%.” (Line 426-430)***

***Additionally, we have made several minor revisions throughout other sections of the manuscript to grasp this key theme. These changes are relatively scattered and are not listed individually here, but all are aimed at improving the overall quality and readability of the manuscript.]***

**Comment 2：**Abstract section and lines 321-323: mildly aged households (60-69) were identified as the main agricultural labor force, accounting for 45.8%; “Analysis of household characteristics showed that the mildly aged households were the primary labor force in rural "areas, accounting for 45.8%” Your expression is incorrect. The statement that the family is the labor force is incorrect. Please revise.

***[Answer: Thanks for your constructive comment.*** ***We deeply apologize for this unreasonable statement. Therefore, we have made modifications and adjustments to the relevant parts that appear in the manuscript (Line 33-34, 352-354, 502-504, 534-537). The detailed descriptions are as follows:***

***“Members of mildly aged households (60-69) were identified as the main agricultural labor force, accounting for 46.6%.” (Line 33-34)***

***The basic information of households of different age groups is shown in Table 3. Analysis of household characteristics showed that 45.8% of households are mildly aged households, and their members have become the primary labor force in rural areas, accounting for 46.6%. (Line 352-354)***

***The results showed that 45.8% of the surveyed households were classified as mildly aged households, whose members have become the primary labor force in rural areas, accounting for 46.6%. (Line 502-504)***

***Analysis of the questionnaire data showed that members of mildly aged households have become the primary labor force in rural agricultural production. (Line 534-537)]***

**Comment 3：**In your variable setting section, it seems that aging should be an explanatory variable rather than a key variable, and abandonment is also a key variable in this study.

***[Answer: Thanks for your constructive comment.*** ***We sincerely apologize for the confusion caused by the inaccurate classification and description of the variables in the original manuscript. Following your suggestions, we have revised the description in variable selection (Line 268-269, 272-273, 279). Specifically, the variable reflecting farmland abandonment is now clearly defined as the dependent variable; the variables representing population aging are identified as the key explanatory variables, as they constitute the theoretical focus of our study; other factors that may influence farmland abandonment are now uniformly referred to as other explanatory variables, including farmer features, the utilization characteristics of farmland, and local socioeconomic and natural environment. The detailed revisions are as follows:***

***“Dependent variable. To reflect the problem of farmland abandonment, the dependent variable in this study is whether farmland is abandoned.” (Line 268 - 269)***

***“Key explanatory variables. The key explanatory variable is the degree of rural population aging and this study will measure it at both regional and household levels.” (Line 272 - 273)***

***“Other explanatory variables.” (Line 279)]***

**Comment 4：**In your line 423: the aging population in rural areas serves as an endogenous driving force behind farmland abandonment. The description of endogenous motivation is inaccurate. It is suggested to be modified.

***[Answer: Thanks for your constructive suggestion and we agree with your comment. This is indeed something worth consolidating.*** ***As we have made adjustments to this section and also recognize that the sentence in question is not appropriately expressed, we have decided to remove it.]***

**Comment 5：**Lines 471-473: Your policy recommendations should be based on your research conclusions. It is not advisable to add any other content here as the description is rather confusing. It is recommended to reorganize them.

***[Answer: Thanks for your constructive suggestion and we agree with your comment. This is indeed something worth consolidating.*** ***We deeply apologize for this unreasonable statement. Therefore, we have made modifications and adjustments to the relevant parts (Line 496 - 501). The detailed revisions are as follows:***

***“These natural constraints, coupled with the pronounced aging of the rural population, contribute to a more complex and severe farmland abandonment issue compared to other regions. Consequently, developing effective strategies for managing abandoned farmland in such areas holds significant practical relevance. In addressing these challenges, it is essential to consider not only food security concerns but also the unique ecological functions that abandoned farmland may serve [72, 73].” (Line 496 - 501)]***

**Comment 6：**The introduction should highlight your research topic, namely two aspects: aging and abandonment. If the current background of aging and abandonment in China is not described clearly, the urgency and reality of the research should be emphasized. It is suggested to supplement relevant current background data. The introduction of the problem was rather abrupt.

***[Answer: Thank you for your constructive suggestion and we fully agree with your comment. The original introduction did not adequately convey the importance and urgency of this research, which indeed warranted improvement. Accordingly, in the revised version of the Introduction, we have added relevant statements concerning abandoned farmland (Line 55- 66) and population aging (Line 69- 82), and emphasized both the urgency and reality of the research. We have also improved the relevant expressions in the Introduction regarding the selection of Ulanqab in the FPENC as the research object (Line 96-102, 105-107). The detailed revisions are as follows:***

***“******Previous studies have indicated that from 1992 to 2020, the global area of abandoned farmland reached 101 million hectares [2], with abandonment observed to varying degrees across Europe [3, 4], North America [5], and Asia [6, 7]. However, in China, with the rapid advancement of industrialization and urbanization, the large-scale outflow of agricultural labor and the continuous decline in agricultural income have contributed to the ongoing expansion of abandoned farmland, which has become progressively more prominent and severe [8]. Abandoned farmland has affected rural livelihoods and agricultural practices [9], while also impacted ecosystem structures [10]and biodiversity patterns [11, 12], thereby exerting significant impacts on socio-economic development and the ecological environment [13, 14]. As a populous country and a major agricultural producer, the abandonment of farmland in China has been recognized as a potential threat to national food security [15, 16]. Therefore, there is an urgent need to address this issue and to formulate context-specific mitigation strategies.” (Introduction, Line 55 - 66)***

***“Among these factors, population aging has increasingly been recognized as a critical influencing variable [19]. With declining birth rates and increasing life expectancy, population aging has become an increasingly serious social issue [20], particularly in rural China where it is more pronounced [21]. According to the Seventh National Population Census in 2020 in China, the population aged 60 and above was approximately 264 million, accounting for 18.7% of the total population. In rural areas, 29.4% of agricultural workers fell into this age group. The rising proportion of elderly individuals, along with diminished labor capacity and declining willingness to manage farmland, has intensified farmland abandonment [19]. However, the relationship between aging and land abandonment remains contested. Some studies have established a positive association between farmers’ age and farmland abandonment in rural areas, particularly in mountainous regions [22-26], while others have found a negative correlation between the age group of 65 and over and the rate of abandonment[27]. These contradictory findings underscore the need for mechanism identification, particularly in region-specific contexts within China, where the pathways of influence and spatial heterogeneity require further investigation.” (Introduction, Line 69 - 82)***

***“Since the 20th century, the FPENC has also experienced more pronounced and severe rural population aging compared to other areas [35]. Compared with previous studies on farmland abandonment [36-38], the ecological vulnerability and accelerating population aging in the FPENC underscore the importance of placing greater emphasis on balancing ecological productivity and land use in addressing farmland abandonment. Therefore, in-depth analyses of the impacts of farmland abandonment in the FPENC with deep population aging are crucial for ensuring regional food security and ecological conservation, and can also provide valuable insights for similar regions in China and globally.” (Introduction, Line 96 - 102)***

***“In this study, we chose Ulanqab in the central part of the FPENC, which is characterized by pronounced population aging and obvious phenomenon of abandoned farmland, as a typical research area.” (Introduction, Line 105 - 107)]***

**Comment 7：**In the section of theoretical analysis and research hypotheses, there are two issues: 1. The problem has not been thoroughly explained. Why does aging accelerate the abandonment of farmland and make it more likely for young families to abandon farmland? A limited theoretical exposition is not sufficient to arrive at your research hypothesis 123. The theoretical derivation of the assumption that there are differences within elderly families and that severely elderly people are more likely to accelerate abandonment is insufficient. It is suggested that the text be further polished.

***[Answer: Thank you for your constructive suggestion. It is indeed worth supplementing and further refining. We have reorganized the theoretical framework to enhance its coherence and explanatory power.*** ***Specifically, we have analyzed how aging accelerates farmland abandonment by distinguishing the behavioral logic of both elderly and young households. Additionally, we have improved and supplemented the discussion on the heterogeneity of farmland abandonment behaviors among elderly households, highlighting the differences in farmland use decisions between mildly and severely aged households. These revisions can be found in the theoretical framework and research hypotheses (lines 114-122, 124, 127-129, 133-160).***

***“Drawing upon the theory of economies of scale in land use and the law of diminishing returns [41], household farmland-use behavior can be interpreted as a rational decision aimed at maximizing benefits under constraints of resource endowment [22, 42]. When the scale of household farmland reaches an optimal threshold (A_2_), per capita net income is maximized. if the farmland area is below the optimal threshold (A < A_2_), income maximization has not been achieved, and households tend to expand their farmland to improve income. Conversely, if the farmland area exceeds the optimal threshold (A > A_2_), households tend to sublet or abandon farmland to reduce operational scale and restore optimal efficiency. For farmland with low subletting value or poor quality, abandonment is often considered inevitable (Fig 1).” (The theoretical framework and research hypotheses, Line 114 - 122)***

***“However, in practice, most households do not exhibit fully rational behavior.” (Line 124)***

***“In this context, household characteristics, particularly age structure, significantly influence their farmland use decisions, thereby contributing to differences in the driving mechanisms of farmland abandonment.” (Line 127 - 129)***

***“Compared to young households, elderly households generally exhibit reduced physical strength and economic inputs, and they experience a diminished capacity for learning and lower adoption of technology [22, 49]. These constraints collectively reduce the optimal farmland management scale for elderly farmers (A_old_), which is lower than that of younger households (A_young_). When the actual farmland area for elderly farmers exceeds the optimal scale, it becomes difficult to achieve maximum agricultural income and thereby increasing the risk of farmland abandonment (Fig 2b). Furthermore, many elderly households exhibit low willingness to continue farming due to limited agricultural income and high labor intensity. Therefore, abandonment often becomes a passive and unavoidable choice. This study proposes Hypothesis 1: age will exacerbate farmland abandonment in elderly households.***

***With the progression of population aging, elderly members often lose the physical capacity to continue farming, prompting the transfer of farmland management responsibilities to young members. However, these younger members face considerable opportunity costs: compared to the relatively low returns from cultivating farmland, urban employment offers more attractive economic prospects. Consequently, young households are more inclined to abandon farmland. Therefore, the following research hypothesis is proposed:***

***Hypothesis 2: the driving factors of farmland abandonment differ significantly across different age groups of households.***

***Furthermore, elderly households are not a homogeneous group and should be further differentiated. Although some households have entered the aging stage, they retain a degree of agricultural production capacity due to relatively good physical health and available labor. These are referred to as “mildly aged households” (Fig 2a). They choose to return to rural areas for farming because of limited urban employment opportunities [50], and may maintain existing farmland use or even continue agricultural production through small-scale reinvestment. In contrast, severely aged households are more likely to abandon farmland due to diminished labor capacity, and a lack of motivation or ability to engage in farming activities. Therefore, this study proposes Hypothesis 3: an increase in the proportion of the mildly aged population will alleviate farmland abandonment to some extent.” (Line 133 - 160)]***
